# The acute vestibular syndrome: prevalence of new hearing loss and its diagnostic value

**DOI:** 10.1007/s00405-023-08296-z

**Published:** 2023-11-09

**Authors:** Moritz von Werdt, Athanasia Korda, Ewa Zamaro, Franca Wagner, Martin Kompis, Marco D. Caversaccio, Georgios Mantokoudis

**Affiliations:** 1 Department of Otorhinolaryngology, Head and Neck Surgery, Inselspital, University Hospital Bern and University of Bern, 3010 Bern, Switzerland; 2 University Institute of Diagnostic and Interventional Neuroradiology, Inselspital, University Hospital Bern and University of Bern, Bern, Switzerland

**Keywords:** Acute vestibular syndrome, Hearing loss, Vestibular stroke, HINTS plus

## Abstract

**Objectives:**

To assess the prevalence of new hearing losses in patients with acute vestibular syndrome (AVS) and to start to evaluate its diagnostic value for the differentiation between peripheral and central causes.

**Design:**

We performed a cross-sectional prospective study in AVS patients presenting to our Emergency Department (ED) from February 2015 to November 2020. All patients received an MRI, Head-impulse test, Nystagmus test and Test of skew (‘HINTS’), caloric testing and a pure-tone audiometry.

**Results:**

We assessed 71 AVS patients, 17 of whom had a central and 54 a peripheral cause of dizziness. 12.7% had an objective hearing loss. ‘HINTS’ had an accuracy of 78.9% to diagnose stroke, whereas ‘HINTS’ plus audiometry 73.2%. ‘HINTS’ sensitivity was 82.4% and specificity 77.8% compared to ‘HINTS’ plus audiometry showing a sensitivity of 82.4% and specificity of 70.4%. The four patients with stroke and minor stroke had all central ‘HINTS’. 55% of the patients did not perceive their new unilateral hearing loss.

**Conclusions:**

We found that almost one-eighth of the AVS patients had a new onset of hearing loss and only half had self-reported it. ‘HINTS’ plus audiometry proved to be less accurate to diagnose a central cause than ‘HINTS’ alone. Audiometry offered little diagnostic accuracy to detect strokes in the ED but might be useful to objectify a new hearing loss that was underestimated in the acute phase. Complete hearing loss should be considered a red flag, as three in four patients suffered from a central cause.

## Introduction

Acute vestibular syndrome (AVS) consists of sudden onset of vertigo nausea and/or postural instability and head motion intolerance combined with spontaneous nystagmus for at least 24 h [1, 2]. Sudden sensorineural hearing loss (SSNHL) which is defined as a sudden loss of 30 dB or greater in at least three sequential frequencies might occur isolated or in combination with vertigo and dizziness [3–5]. The underlying etiology and its therapy is similar for both entities [4, 6–8]. It has been suggested that AVS and concomitant SSNHL is a central sign, caused by ischemia through the labyrinthine end artery and consequently causing labyrinthine infarction [2, 9]. The labyrinthine artery is often involved in posterior circulation strokes, such as basilar artery and AICA strokes, which leads to a hearing loss in 60–90% of cases [10–13]. Vertigo and hearing loss has also been described as a warning sign of impending AICA infarction [10]. A large proportion of patients with AICA strokes (7.4%) had a previous episode of vertigo and hearing loss with normal brain MRI prior to the ED presentation [14]. In another study, 9/29 (30%) patients with AICA infarction reported audiovestibular loss up to 10 days prior to the stroke [11]. AVS and SSNHL, however, occur frequently as a benign labyrinthitis with possible viral/autoimmune etiology [6]. ‘HINTS’ (Head Impulse-Nystagmus-Test of Skew) is an established bedside test with great accuracy to differentiate central from peripheral cause of AVS [2, 15]. Adding a bedside hearing evaluation, mostly finger rubbing next to patient’s ears, asking for asymmetry, coined the term ‘HINTS’ plus [2, 9, 16]. Analyzing ‘HINTS’ “plus”, Newman-Toker et al. showed an increase in sensitivity for stroke, but slight decrease in specificity, postulating hearing loss to be a central sign in AVS. Several other authors have supported this [9–11, 17, 18]. The prevalence of vertigo/imbalance in SSNHL has been reported to be 31% [19]. To our best knowledge, the prevalence of hearing loss in AVS patients has not been described yet. In this prospective cross-sectional study, we sought to assess the prevalence of new hearing loss in AVS patients with audiometry and to evaluate its diagnostic accuracy to detect strokes.

## Materials and methods

### Patient characteristics

**Fig. 1:**
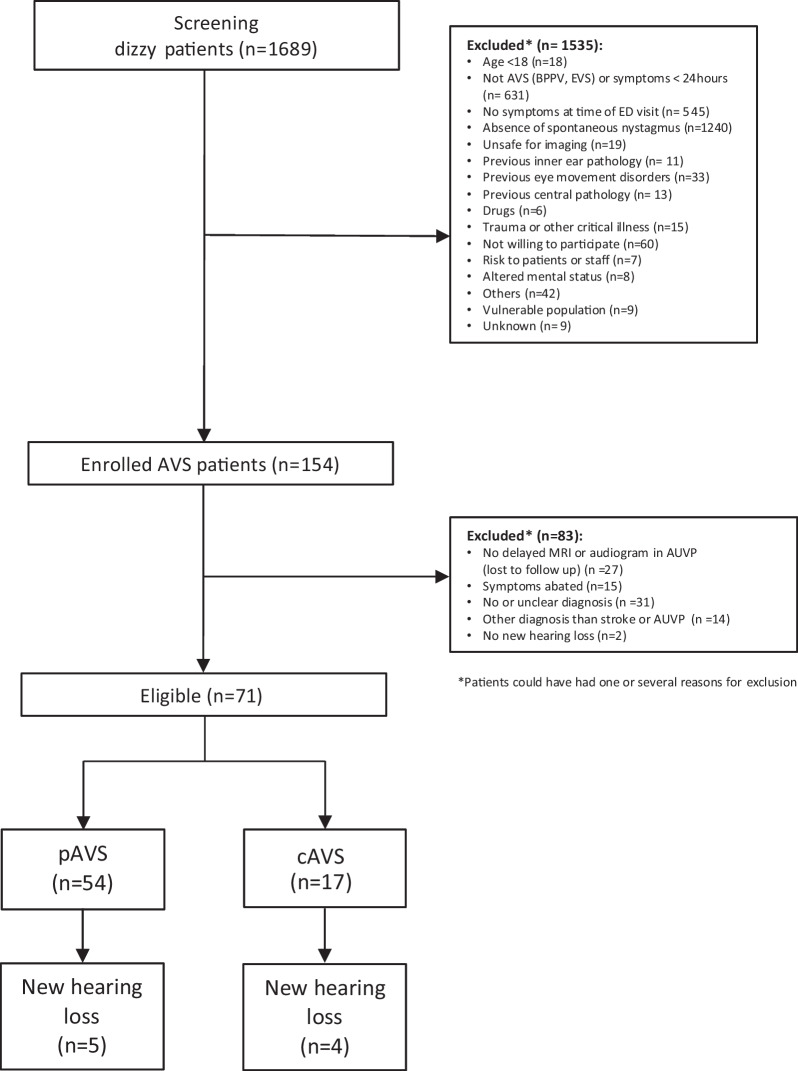
Flowchart showing inclusions and exclusions of dizzy patients in the ED

We included for this study patients older than 18 years with peripheral AVS (pAVS, vestibular neuritis or labyrinthitis) or stroke (central AVS, cAVS) as part of a prospective cross-sectional study in the emergency department (ED) (DETECT—Dizziness Evaluation Tool for Emergent clinical Triage) during 02/2015 to 11/2020. The population and inclusion criteria of the DETECT study have been previously described [20]. Patients with middle ear pathologies (otosclerosis, middle ear effusion) were excluded. We enrolled 154 AVS patients and excluded 83 patients, since they received no MRI and/or audiogram (*n* = 27), symptoms abated at the time of examination (*n* = 15), had no or unclear diagnosis (*n* = 31), had another diagnosis than stroke or pAVS, and/or had a pre-existing unilateral hearing loss (*n* = 2) (Fig. 1). Trained research staff screened dizzy patients during office hours and emergency physicians outside of office hours using Frenzel goggles (blocked visual fixation) to examine for nystagmus in all gaze directions. Trained neurotology physicians examined all included patients. All included patients received ‘HINTS’, calorics and at least an air conduction audiogram within 72 h after symptom onset.

### Patient classification

We classified patients into central or peripheral causes of AVS based on imaging, neurootological tests, and clinical examination. The delayed MRI served as a gold standard for stroke detection (delayed MRI at least 48 h after symptom onset with the following sequences: diffusion weighted imaging, axial T1-weighted (T1w) and T2-weighted (T2w) sequences for the whole brain, an SWI (susceptibility weighted imaging) sequences covering the whole brain, a coronal whole brain FLAIR sequences, an axial T2w covering the temporal bone, a 3D axial CISS above the temporal bone at the level of the IAC, and an intracranial 3D-TOF (time of flight) MR angiography). Patients with (1) a symmetrical neurootological examination (bilateral normal or bilateral abnormal caloric testing), and/or (2) central neurological signs and (3) negative MRI were classified as a minor stroke (cAVS). Patients with confirmed stroke in the MRI were classified as major stroke (cAVS). All cAVS patients received diagnostic stroke workup and treatment in the ED or stroke unit.

Patients with peripheral symptoms in the neurootological examination (mixed horizontal-torsional nystagmus beating away from the lesion side, no other focal neurologic signs) and a pathologic bithermal caloric test (> 20% asymmetry) based on our laboratory normative values and negative MRI, were classified as pAVS. Classification was done by expert consensus.

All patients received at least an air conduction audiometry with calibrated headphones in a sound proofed cabin.

New hearing loss was defined as newly detected hearing loss in audiometry in the ipsilateral ear of the affected side (stroke/pAVS) compared to the better hearing ear in each patient. Cutoff for hearing loss was a mean asymmetry of 30 dB hearing level (HL) or more over three consecutive frequencies [3–5]. We calculated the difference of hearing loss in dB in (pure-tone average (PTA) over the frequencies of 500, 1000, 2000, and 4000 Hz) of the affected side in air conduction to the unaffected side (asymmetryPTA, aPTA).

### Statistics

All statistics were performed using SPSS and R statistical software (IBM SPSS Statistics for Windows, Version 25.0. Armonk, NY: IBM Corp., R 4.2.2., R Core Team 2021 and ‘ggplot’ package). We used cross-tabulations to assess specificity, sensitivity, and accuracy for ‘HINTS’ and the combination with audiometry. We calculated the receiver characteristic curves (ROC) using aPTA and PTA as predictors for a central cause of AVS. Cohen’s Kappa was calculated for the assessment of agreement between subjective and objective hearing loss.

### Ethical considerations

All enrolled patients gave written consent. The local ethics committee (IRB) approved this study (KEK # 047/14).

## Results

We assessed 71 patients (Fig. 1) with AVS, 40 of which were male and 31 female. The mean age was 55 years (range 20–88 years). 54 patients had peripheral and 17 had a central cause of AVS. Seven were diagnosed with a stroke in the MRI (3 PICA strokes, 1 combined AICA and PICA stroke, 1 middle cerebral artery stroke, 1 anterior cerebral artery stroke, and 1 vertebral artery stroke). Nine were diagnosed as minor strokes with abnormal clinical findings but normal delayed MRI and normal vestibular function (symmetric calorics) and one patient had a herpes zoster encephalitis presenting with AVS.

### Prevalence of hearing loss in AVS

**Fig. 2:**
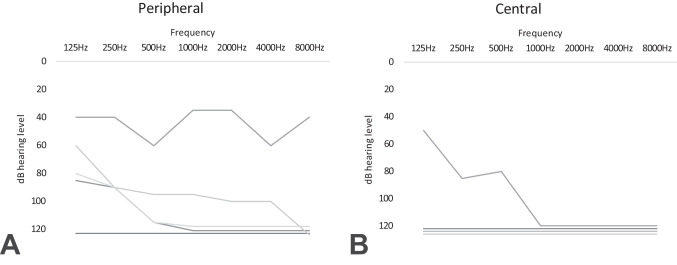
Air conduction audiometry for the affected side. *X*-axis: frequency in Hz, *Y*-axis: decibel hearing level (dB HL). Overall, nine patients suffered from a new onset of hearing loss and vertigo. **A** Audiometry from five patients with pAVS and **B** four patients with a stroke/minor stroke

The prevalence of a new asymmetric hearing loss in AVS patients was 12.7% (9/71, Table 1). Four out of 17 (23.5%) patients with central AVS had a new hearing loss (Fig. 2B) compared to 5 out of 54 (9.3%) with peripheral AVS (Fig. 2A). Complete unilateral hearing loss occurred in both groups (peripheral 1/5, central 3/4). Cohens Kappa for concordance of subjective vs objective hearing loss was 0.532 (moderate agreement). Only 5/9 (55%) of patients with a significant unilateral hearing loss (> 30 dB PTA) self-reported their hearing loss.

**Table 1:** Patient characteristics with new hearing loss

#	Sex	Age	Diagnosis	Affected side	Caloric asymmetry	aPTA	PTA	‘HINTS’	cvRF	Neuro-logical signs	ABCD2 Score
1	f	78	pAVS	Right	Yes	103	120	Peripheral	1 (HT)	No	4
2	m	69	Minor stroke	Left	No	66	100	Central	1 (HT, smoking)	No	4
3	f	75	pAVS	Left	Yes	75	98	Peripheral	4 (HT, Lipid, TIA)	No	4
4	m	68	Minor stroke	Left	No	92.5	120	Central	0	No	3
5	f	54	pAVS	Left	Yes	111	120	Peripheral	0	No	2
6	f	46	Minor stroke	Right	No	116	125	Central	1 (OSAS)	No	2
7	f	67	pAVS	Right	Yes	81	120	Central	2 (HT, DM)	No	5
8	m	71	Stroke	Right		65	113	Central	3 (Lipid, HT, CVI)	Ataxia, double vision,	4
9	m	20	pAVS	Left	Yes	35	51	Peripheral	1 (smoking)	No	2

*pAVS* peripheral acute vestibular syndrome, *HT* hypertension, *TIA* transient ischemic attack, *OSAS* obstructive sleep apnea syndrome, *DM* diabetes mellitus, *CVI* cerebrovascular insult, *HINTS* Head-Impulse-Nystagmus-Test-of-Skew, *aPTA* Mean hearing level difference compared to the contralateral, unaffected ear, *PTA* mean pure-tone audiometry of the affected ear, *cvRF* cardiovascular risk factor, *ABCD2* Age, blood pressure, clinical features of TIA, diabetes, duration of symptoms

### Hearing loss severity and patterns in AVS

We did not observe any specific patterns in audiograms (high-frequency, low-frequency, and pancochlear hearing loss) for either central or peripheral causes (Fig. 2Aperipheral, Bcentral). Dizzy patients with a hearing loss showed large variations in severity with PTAs ranging from 48 to 120 dB hearing level. There was a large overlap for central or peripheral AVS with normal hearing; however, the proportion of patients with an asymmetric hearing loss > 60 dB PTA and central AVS was higher. None of the patients with central AVS had hearing loss in the low-moderate range. Analysis of logistic regression variables for aPTA, age, and gender were not significant.

### Accuracy of ‘HINTS plus audiometry’

**Fig. 3:**
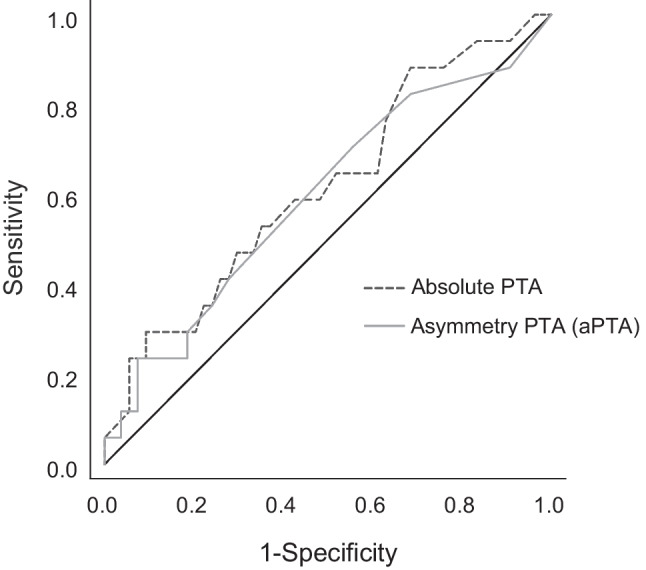
Receiver characteristic curve showing the sensitivity and specificity for each test, aPTA (difference between better hearing ear and the ear with new hearing loss) and PTA (pure-tone average of the affected ear). The diagonal line represents a likelihood ratio of 1 at all thresholds, indicating an ineffective test

The area under the curve in the receiver characteristic curve (ROC, Fig. 3) was 0.596 (*p* = 0.082) for aPTA and 0.617 (*p* = 0.079) for the mean hearing level (PTA). There was no significant cut-off to differentiate peripheral from central cause in ROC for either measurement (Fig. 3). Measured hearing loss was not attributed to be more prevalent in central or peripheral cause. Clinical ‘HINTS’ had an accuracy in detecting central cause of 78.9% vs ‘HINTS’ plus audiometry’ 73.2%. ‘HINTS’ sensitivity was 82.4% and specificity 77.8% compared to ‘HINTS plus audiometry’ showing a sensitivity of 82.4% and specificity of 70.4% (Table 2). The four patients with hearing loss and central AVS (stroke and minor stroke) had all central ‘HINTS’.

**Table 2:** Cross-tabulation analysis

Diagnostic test		Diagnosis		
		Peripheral	Central	Total
‘HINTS’	Peripheral	42 (TN)	3 (FN)	45
	Central	12 (FP)	14 (TP)	26
‘HINTS’ + audiometry	Peripheral	38 (TN)	3 (FN)	41
	Central	16 (FP)	14 (TP)	30
	Total	54	17	71

*TN* true negative, *FN* false negative, *FP* false positive, *TP* true positive

## Discussion

Almost one-eighth of AVS patients in the ED had a new hearing loss, half of which were not self-reported. We performed pure-tone audiometry additionally to ‘HINTS’, which did not increase the accuracy in detection of central AVS. Patients do not reliably report hearing loss in the acute setting, which has been previously reported, suggesting that this might be due to focus on the much more disabling vertigo [21, 22].

### Hearing loss prevalence

The proportion of 76% peripheral vs 23.9% central AVS is similar to the literature (3/4:1/4) [18]. We found a high prevalence of 12.7% in AVS patients with a new single-sided hearing loss. Carmona et al. studied a similar cohort of 114 patients with AVS of which all received an MRI and audiometry. In their results, 72 patients had an acute unilateral vestibulopathy and 42 were diagnosed with stroke. The authors reported a hearing loss prevalence of 40% in patients with AICA strokes and an overall higher prevalence of 23% in AVS [23], since they included more AICA strokes with labyrinthine infarctions. Pogson et al. and Bery and Chang published data from 13 AVS patients with SSNHL, five of which had central lesions (MRI+), one of them with severe hearing loss (PTA 90 dB, lateral pontine infarction) [18, 24].

### Is the labyrinthine artery an end artery?

We found a predominately complete hearing loss across all frequencies which has also been described in stroke patients with labyrinthine infarction [11]. The labyrinthine artery has always been considered an end artery, a branch from the AICA; however, it might also arise from the basilar artery or rarely from the PICA. This anatomical variability suggests that the cochlear blood supply might be influenced by collaterals [25, 26], explaining some reported partial hearing loss (ranging between 33 and 95 dB PTA) and potential recovery of hearing function [11]. Various animal studies with labyrinthine artery occlusions demonstrated that the more peripheral the occlusions occurred (at the level of the inner ear canal), the more cochlear blood flow was compromised [27].

### Hearing loss: a red flag for stroke?

Various studies support that a large proportion of patients with AICA infarctions had previous prodromal symptoms of audiovestibular loss up to 1 month prior to infarction [14, 17]. Chang et al. reported higher stroke rates of 5.5% of patients with vertigo and hearing loss vs patients with vertigo alone (3.9%) [28]. Kim et al. showed similar results in a longitudinal follow-up cohort study with an SSNHL posing an adjusted hazard ratio for SSNHL for ischemic stroke of 1.22 [29].

Although a new hearing loss was found to be a predictor for stroke especially in patients with suspected AICA stroke and false peripheral ‘HINTS’ exam [2, 30], some studies including hearing tests and tuning fork testing did not find any improved accuracy in AICA stroke diagnosis [15, 23]. In our study, performing audiometry as a standalone test in the acute setting in AVS patients was not differentiating central from peripheral causes. The accuracy of ‘HINTS’ plus audiometry was even lower. However, we consider patients with a hearing loss > 60 dB HL being at a higher risk for central AVS, since 75% of patients with hearing loss (> 60 dB) had a stroke/minor stroke. A majority of AVS patients, however, had a peripheral cause of audiovestibular loss, and should receive audiometry to evaluate a potential corticosteroid therapy [5, 31].

### Strength and limitations

We studied 71 patients with AVS who received an audiometry, which offers a higher resolution compared to finger rubbing [32]. We did not have prior audiograms of our patients to consider new hearing loss when comparing hearing levels of the affected side. There might be a bias, since patients did not always notice or report previous unilateral hearing loss. There are reports about AICA strokes affecting hearing on both sides, which could not have been detected without audiometry before symptom onset. There might be a selection bias, since stroke patients did not systematically receive audiometry due to the urgency and prioritization of stroke diagnosis and treatment in the acute setting. In addition, we only included one AICA stroke in our study population, which underestimates the prevalence of hearing loss in central causes of AVS. Hearing loss in the acute phase within the first 24 h after symptom onset might also be due to a vestibular migraine or Menière’s disease; however, we excluded patients whose symptoms abated after 24 h.

### Implications

The prevalence of a new hearing loss in AVS patients is high and warrants a follow-up hearing test, even in patients who do not report it as a chief complaint. In our cohort, audiometry in the acute setting did not help to differentiate peripheral from central etiology; however, it still can be considered a red flag in patients with a severe hearing loss (> 60 dB). Early detection of patients being at risk for stroke (with hearing loss as a prodromal symptom) is crucial. A tuning fork test/finger rubbing test would be an appropriate test in the acute phase.

## Conclusions

We found that almost one-eighth of the AVS patients had a new onset of hearing loss and only half had self-reported it. ‘HINTS’ plus audiometry proved to be less accurate to diagnose a central cause than ‘HINTS’ alone. Pure-tone audiometry offered little diagnostic accuracy in detecting strokes in the ED but might be useful to objectify a new hearing loss that was underestimated in the acute phase. Complete hearing loss should still be considered a red flag, as three in four patients suffered from a central cause.
